# Supplementary material for: Macrophage-Colony-Stimulating Factor Receptor Enhances Prostate Cancer Cell Growth and Aggressiveness In Vitro and In Vivo and Increases Osteopontin Expression
Source: Int J Mol Sci. 2022 Dec 16;23(24):16028. doi: 10.3390/ijms232416028 (PMC9785574; doi:10.3390/ijms232416028)
Supplement: Supplementary file 1 [file ijms-23-16028-s001.zip › Supp Figure S1 Mougel et al.pdf]

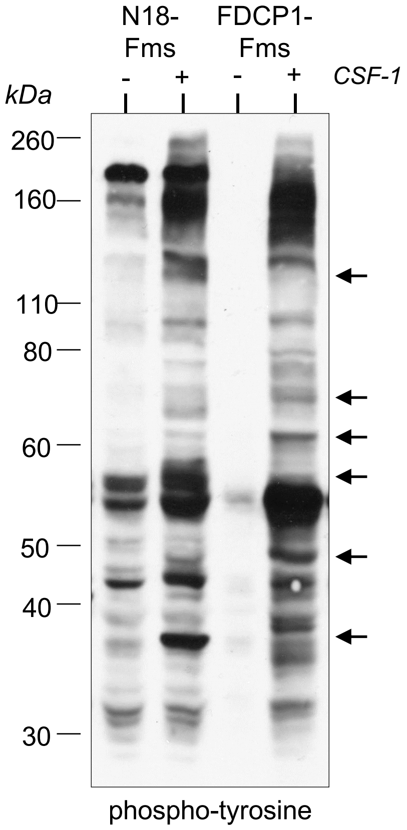

Supplementary Figure S1: Stimulation by CSF-1 of CSF-1R-expressing cells. Immunoblot analysis of protein tyrosine phosphorylation patterns shows differences (arrows) between stimulated myeloid FDCP1-Fms cells and prostate cancer N18-Fms cells.
