# Supplementary material for: Macrophage-Colony-Stimulating Factor Receptor Enhances Prostate Cancer Cell Growth and Aggressiveness In Vitro and In Vivo and Increases Osteopontin Expression
Source: Int J Mol Sci. 2022 Dec 16;23(24):16028. doi: 10.3390/ijms232416028 (PMC9785574; doi:10.3390/ijms232416028)
Supplement: Supplementary file 1 [file ijms-23-16028-s001.zip › supp Figure S2 mougel et al.pdf]

A

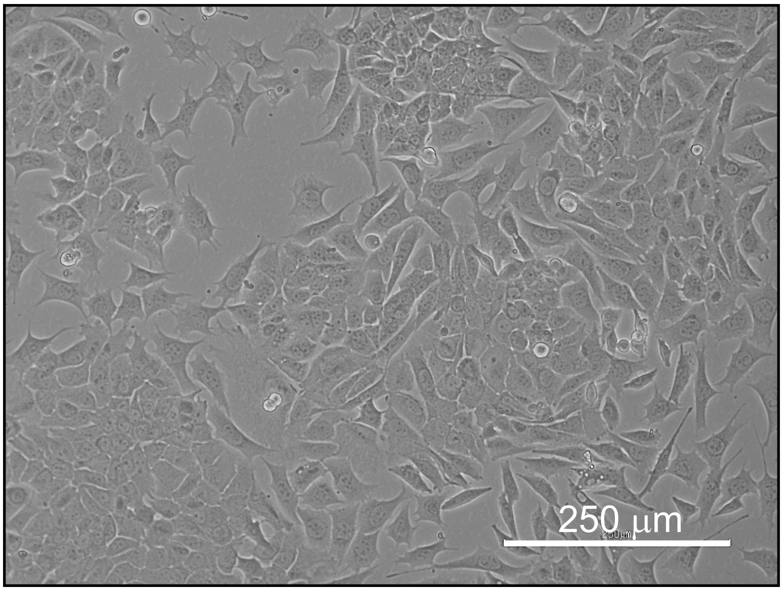


B

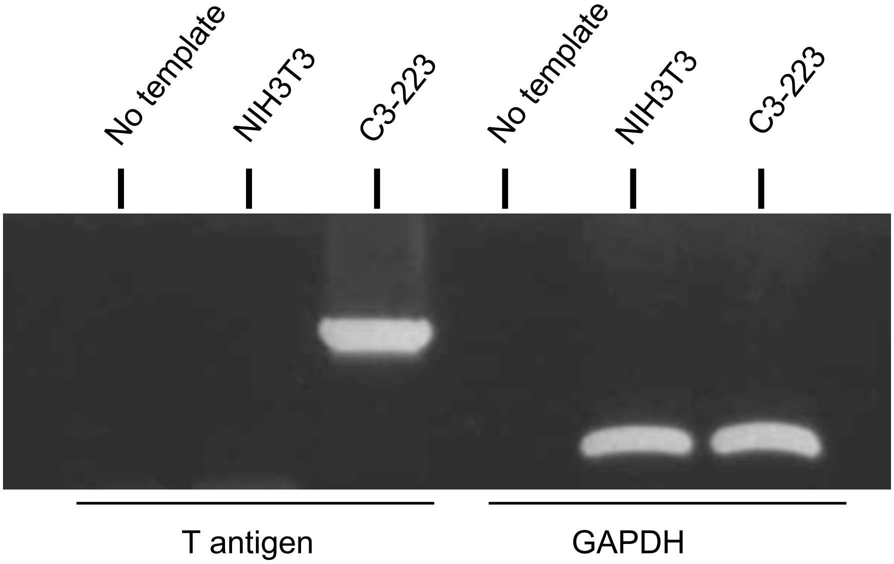


**Supplementary Figure S2**: C3-223 mammary cancer cell line.
(**A**) Representative photograph of murine C3-223 mammary cancer cell line, scale bar, 250µm. (**B**) T-antigen expression was analysed by a single PCR on genomic DNA extracted from murine C3-223 mammary cancer cell line and murine fibroblast NIH 3T3 cell line (as a negative control) with the primer set TA1/TA2 :
(GACCTGTGGCTGAGTTTGCTCA) / (GCTTTATTTGTAACCATTATAAG). GAPDH expression was used as a control of the quality of genomic DNA extraction.
